# Genome sequence of the dimorphic *Dothioraceae* fungal isolate EMM_F3

**DOI:** 10.1128/mra.00396-25

**Published:** 2026-04-20

**Authors:** Beatrice Severance, Sachida Pokhrel, Zachary A. Noel

**Affiliations:** 1 Department of Entomology and Plant Pathology 1383, Auburn, Alabama, USA; University of California Riverside 8790, Riverside, California, USA

**Keywords:** yeast, genome analysis, *Dothidiomycete*

## Abstract

Phyllosphere yeasts are abundant on plant surfaces but understudied. The dimorphic fungal isolate EMM_F3, from *Magnolia grandiflora*, had its genome drafted and assembled. Preliminary phylogenetic analysis places EMM_F3 in the Dothideales order, likely in the *Dothioraceae* family, closely related to *Delphinella strobiligena*.

## ANNOUNCEMENT

The phyllosphere offers the vast surface area for microbial colonization (1–3). Although yeasts in these habitats have historically been understudied (3, 4).

**Fig 1:**
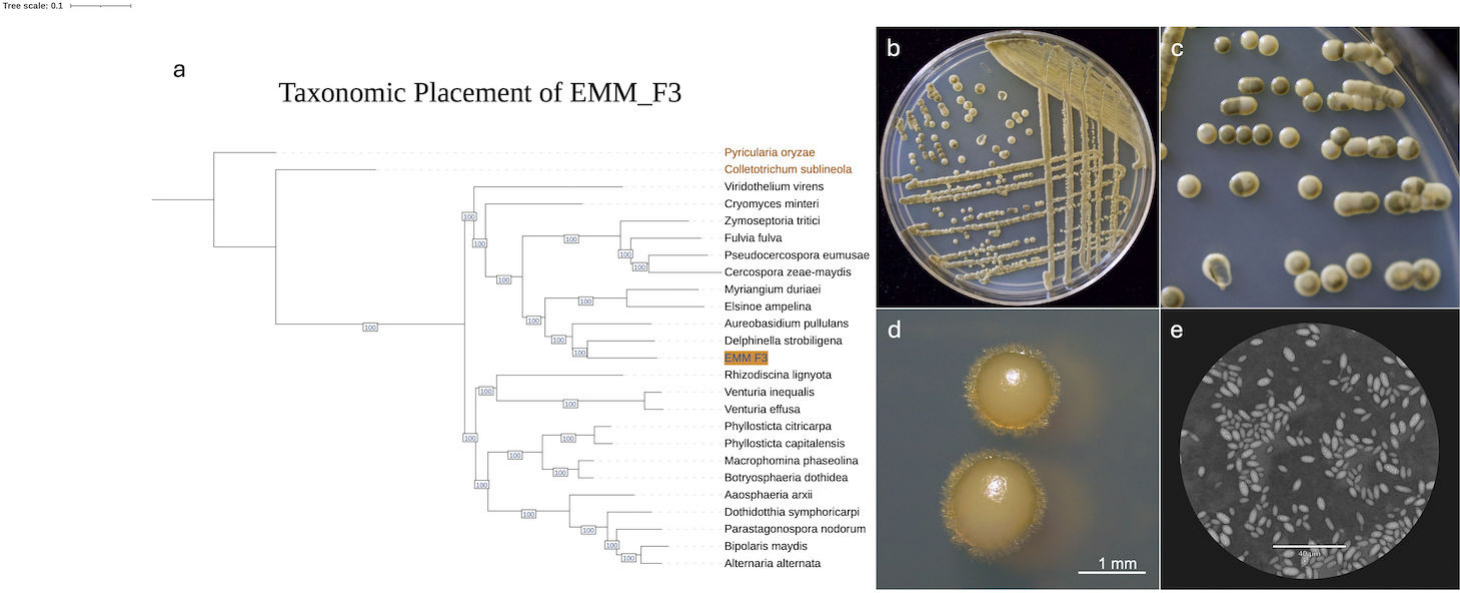
Evolutionary placement and morphological features of EMM_F3. (**a**) Maximum likelihood tree generated using IQ-TREE through the “compare” function of the funannotate program based on 500 single-copy orthologous genes. Outgroups are from the Sordariomycetes class and are displayed in orange text. EMM_F3 is highlighted in orange. Bootstrap values are displayed in blue and are based on 100 iterations. (**b**) Morphological features of EMM_F3 grown on MEA+ for 15 days. (**c**) Close-up image of the colonies in panel **b** showing the melanization of some colonies. (**d**) Photo of EMM_F3 showing hyphal growth extending from the yeast colony. (**e**) microscopic image (40×) of EMM_F3 with India ink for background contrast.

As part of an undergraduate research project, we isolated a yeast (EMM_F3) from the leaf surfaces of *Magnolia grandiflora* in the Auburn University Arboretum. On 21 January 2021, three leaves without visible disease lesions were sampled from three independent Southern Magnolia trees in Auburn, Alabama. The trees were part of the Auburn University Donald E. Davis Arboretum (coordinates: 32.5959, −85.4828). Leaf strips, 4 cm by 10 cm long, were cut with flame-sterilized scissors into a sterile 50 mL tube and vortexed in 1× phosphate-buffered saline for 1 min. One hundred microliters of leaf wash were spread onto Petri dishes containing Difco malt extract agar medium (BD Biosciences, NJ, USA) amended with 1 g of yeast extract (MP Biomedicals, OH, USA), rifampicin (0.01 mg/mL), and chloramphenicol (0.1 mg/mL). Yeast-like colonies were spread onto new malt extract medium to represent a single isolate (Fig. 1b through e). Based on internal transcribed spacer region sequencing (accession number: PV752320), EMM_F3’s closest match was *Dothidea eucalypti* CBS 143417 (NR_156390.1), with an 88% identity over nearly the entire ITS region (98% query coverage). This warranted whole-genome sequencing. EMM_F3 also exhibited dimorphic morphology, changing from yeast form to filamentous growth as it aged on MEA plus yeast extract (Fig. 1d).

Total genomic DNA was extracted via cetyltrimethylammonium bromide extraction (5) and quantified via Qubit. DNA was sequenced on an Illumina NovaSeq 6000, producing 2 × 151 bp reads. Demultiplexing, quality control, and adapter trimming were performed with bcl-convert (v4.0.3). BBDuk (v39.06) was used to remove adapters from raw reads, trim reads, and check for the removal of PhiX (6). FastQC (v0.10.1) assessed read quality (7). Assembly was performed using SPAdes version 3.15.5 (8) and decontaminated using FCS-GX version 0.5.0 (9). QUAST (v5.2.0) assessed assembly statistics, and BUSCO (v5.4.3) determined genome assembly and annotation completeness (10).

EMM_F3’s genome had 2,858,439,152 base pairs (bp) sequenced with a *Q* > 30 (92.14%). The final assembly was 24,166,584 bp long (118× coverage) and contained 123 contigs ≥500 bp. The *N*_50_ was 334,563, and the GC content was 54.39%. A total of 7,730 proteins were predicted from the genome. Additionally, 1,282 complete BUSCOs (97.7%) were matched when choosing Dikarya for comparison. When choosing Dothideomycetes for deeper comparison, 3581 complete BUSCOs (94.6%) were matched. Dothideomycete genomes available on the Joint Genome Institute’s Mycocosym (11) were downloaded and compared phylogenetically to EMM_F3 using IQ-TREE to provide a maximum likelihood tree based on 500 single-copy orthologous genes found with the “funannotate” pipeline (v1.8.13) (12). EMM_F3 fell within a clade containing other melanized yeast-like taxa, *Delphinella strobiligena* and *Aureobasidium pullulans*, demonstrating a morphologically consistent clade (Fig. 1).

Based on these results, EMM_F3 likely represents a novel taxon within the *Dothioraceae*, sharing common ancestry with *Aureobasidium* and *Delphinella*.

## Data Availability

The raw forward and reverse sequences for EMM_F3 are available on the Sequence Read Archive (SRA) under accession number SRP511634, BioProject PRJNA1117874. This Whole Genome Shotgun project has been deposited at DDBJ/ENA/GenBank under accession number JBOGCK000000000. The version described in this paper is version JBOGCK020000000. The ITS sequence accession number for EMM_F3 is PV752320. The pipeline for read processing, assembly, and annotation is located on https://github.com/Beatrice-Severance/Genome_Assembly. Default parameters were used for all software except where otherwise noted. The strain can be obtained by contacting the Noel lab at Auburn University, while it is in the process of being deposited into a culture collection.
